# ADHD symptomatology of children with congenital heart disease 10 years after cardiac surgery: the role of age at operation

**DOI:** 10.1186/s12888-021-03324-w

**Published:** 2021-06-24

**Authors:** Nikoletta R. Czobor, Zsófia Ocsovszky, György Roth, Szabolcs Takács, Márta Csabai, Edgár Székely, János Gál, Andrea Székely, Barna Konkolÿ Thege

**Affiliations:** 1 School of Doctoral Studies, Semmelweis University, Budapest, Hungary; 2 Department of Anaesthesiology and Intensive Care, Medical Centre of Hungarian Defence Forces, Budapest, Hungary; 3 Department of Personality, Clinical and Health Psychology, Institute of Psychology, University of Szeged, Szeged, Hungary; 4 Department of Paediatric Cardiology, Gottsegen György Hungarian Institute of Cardiology, Budapest, Hungary; 5 Institute of Psychology, Károli Gáspár University, Budapest, Hungary; 6 Department of Anaesthesiology and Intensive Care, Semmelweis University, Budapest, Hungary; 7 Department of Paediatric Anaesthesiology and Intensive Care, Gottsegen György Hungarian Institute of Cardiology, Budapest, Hungary; 8 Waypoint Centre for Mental Health Care, Waypoint Research Institute, Penetanguishene, ON Canada; 9 Department of Psychiatry, University of Toronto, Toronto, ON Canada

**Keywords:** Congenital heart disease, Pediatric cardiac surgery, Attention-deficit hyperactivity disorder, Age-related differences

## Abstract

**Background:**

The aim of the present study was to investigate the differences in ADHD symptomatology between healthy controls and children who underwent cardiac surgery at different ages.

**Methods:**

Altogether, 133 children (54 patients with congenital heart disease undergoing first cardiac surgery under 3 years of age, 26 operated at the age of 3 or later, and 53 healthy controls) were examined. Patients completed the Youth Self Report (YSR), while their parents completed the Child Behaviour Checklist (CBCL) and the ADHD Rating Scale-IV.

**Results:**

Children receiving surgery for the first time under the age of 3 years were more likely diagnosed with cyanotic type malformation and have undergone to a greater number of operations. However, ADHD symptoms of those treated surgically at or above 3 years of age were more severe than that of the control group or those who were treated surgically at a younger age. The control group and those treated surgically below the age of three did not differ across any of the ADHD symptom severity indicators.

**Conclusions:**

The age at the time of cardiac surgery might be associated with later ADHD symptom severity – with lower age at operation associated with better outcomes. Further, adequately powered studies are needed to confirm these exploratory findings and investigate the moderators of this relationship.

**Supplementary Information:**

The online version contains supplementary material available at 10.1186/s12888-021-03324-w.

## Background

In recent years, improvements in medical treatments, non-invasive imaging, surgical techniques, as well as advances in cardiopulmonary bypass surgery and pediatric intensive care have allowed more than 90% of children born with congenital heart disease (CHD) to reach adulthood. The overall mortality of this patient population has decreased by 31% over the last 20 years [1]. However, after early surgical corrections, physically healthy grown-up survivors may still face the consequences of the neuronal and psychological injuries they might have suffered during the perioperative period [2]. Cardiac surgery and perioperative complications also have been linked to dysfunctional behaviors and psychological abnormalities such as attention-deficit/hyperactivity-disorder (ADHD) [2–4].

Even though the etiology of ADHD is multifactorial, several studies reported a significantly higher morbidity for this disorder among cardiac surgery patients, especially after aortic arch repairs and corrections of great artery transposition [5, 6]. Children with congenital heart disease have been reported to be at a 30% higher risk for inattention and hyperactivity disorder compared to healthy individuals. Importantly, while nearly half of the surgically treated patients need remedial school services when reaching adolescence, cardiac patients’ ADHD symptoms are often under-diagnosed and thus under-treated [7].

Studies have shown, that adolescents with different cardiac anomalies (single ventricle CHD, TGA, tetralogy of Fallot) have a threefold inreased risk for higher psychiatric morbidity, including attention deficit and hyperactivity disorder, compared to the healthy population [8]. Children with complex CHD have abnormal cerebral blood flow wich is already altered during pregnancy and in the peripartum periods, potentially causing delays in brain development. Additional hypoxaemia due to postponed correction of cardiac defects seems to increase the risk for attentional dysfunction, hyperactivity and it is considered responsible for further damage to the highly oxygen-sensitive regions of the prefrontal cortex [9]. Further risk factors for the development of ADHD include lower birth weight, longer duration of deep hypothermic circulatory arrest, male gender, and repeated surgeries [2].

Previous studies have shown that the risk of developing ADHD in adolescence is significantly higher in cardiac surgery patients compared to healthy individuals. The symptoms may worsen by repeated exposure to anesthetics and surgery-induced stress and inflammatory responses [10]. The literature suggests that hypoxemia – caused by cyanotic heart defects as hypoplastic left-heart syndrome or dextro-transposition of the great arteries – increases the risk of attention deficits, a tendency that was not observable in cases of acyanotic malformations [2, 9]. Other studies however, describe the same neurodevelopmental deterioration in patients with acyanotic heart defects; in these cases though, other cognitive challenges and not attention deficit was the leading symptom [11, 12]. Further studies report that cyanotic heart defects (such as transposition of the great arteries or tetralogy of Fallot) do not increase the incidence of future behavioral disorders in the absence of other risk factors [13].

Recently, it was also suggested that single or repeated anesthesia itself could be a risk factor for the development of ADHD symptoms independently of the surgery performed [14, 15]. Studies specific to neonatal care came to similar conclusions when investigating the effects of different types of cardiac surgery [16, 17]. Patients with severe congenital malformation usually receive palliative surgery as first-line therapy resulting in the partial restoration of fetal circulation. This intervention is needed to promote organ maturation in order to survive complex, high-risk corrective surgeries later. These children usually live with an incomplete circulation for 1–2 years, a potential risk factor for future neuropsychiatric and behavioral disorders.

Unfortunately, there is no clear medical protocol regarding intraoperative cerebral protection. Different neuroprotective operative management strategies, such as the use of deep hypothermic circulatory arrest, may have less influence on behavioral outcomes than had been assumed previously. Studies suggest that the prevalence of ADHD in pediatric cardiac populations remains higher than in the normal population even if operations are conducted in deep hypothermic circulatory arrest, a finding which may point to the role of non-operation-related factors in the development of ADHD [5]. In line with this assumption, other studies showed that genetic abnormalities seem to have an impact on the neurodevelopment of neonatal cardiac surgery patients [18]. Other factors such as complexity of the heart disease or additional genetic malformations [19] or even the patient’s age at surgery may also be significant predictors for psycho-behavioral outcomes.

To date, studies examining the association between ADHD symptomatology and cardiac surgery in children have largely ignored the age of patients at operation and / or did not include healthy individuals as a control group. The primary aim of this study, therefore, was to investigate the potential relationship between later ADHD symptoms and the timing of first cardiac surgery in relation to the age of children (before versus at or after 3 years of age, representing an important milestone in brain development; cf. [20]). Further, we also aimed to compare the ADHD symptomatology of those undergoing cardiac surgery with that of a control group without surgery. Given that extant studies have mostly relied on parent reports, our aim was to investigate the relationship between ADHD symptomatology and age at cardiac operation considering both collateral (parent) and self-reported (pediatric cardiac patient) data.

## Methods

### Sample and procedure

All procedures performed in this study were in accordance with institutional guidelines and the 1964 Helsinki declaration and its later amendments. The study protocol was approved by the Hungarian Medical Research Council (approval number: TUKEB 190/2008; 696–1/2014/EKU 461/2014) and was performed upon participants and parents giving informed consent. Altogether, 133 children were examined: 54 patients with congenital heart disease undergoing first cardiac surgery under 3 years of age, 26 operated at the age of 3 or later, and 53 healthy controls. Detailed demographic data about the study groups are reported in Table 1.

**Table 1:** Demographic, perioperative, and ADHD-related characteristics of the sample

	Control group (*N* = 53)		Surgically treated (*N* = 80)				Test statistic^a^	p	Effect size
			under 3 years of age (*N* = 54)		at / above 3 years of age (*N* = 26)				
	Mean/N	SD/%	Mean/N	SD/%	Mean/N	SD/%			
Age at survey (years)	11.79	3.65	11.37	3.06	14.58	3.42	F = 8.44	< 0.001	η^2^ = 0.113
Sex (N)							χ^2^ = 0.51	0.774	Cramer’s V = 0.062
Male	24	45.28	26	48.1	14	53.8			
Female	29	54.71	28	51.8	12	46.2			
YSR_AD_	2.49	2.18	2.72	2.41	4.31	3.73	F = 4.463	0.013	η^2^ = 0.064
CBCL_AD_	2.02	2.36	2.33	3.05	3.38	3.79	F = 1.89	0.156	η^2^ = 0.028
Diagnosable severity of ADHD symptomatology based on CBCL_AD_ (N)	2	3.77	3	5.88	3	12.00	χ^2^ = 1.99	0.370	Cramer’s V = 0.124
ADHD RS_IA_	2.83	3.34	2.96	4.28	7.73	8.30	F = 9.716	< 0.001	η^2^ = 0.130
ADHD RS_HI_	2.72	3.14	2.61	4.17	6.62	7.34	F = 7.640	0.001	η^2^ = 0.105
ADHD RS_TOT_	5.68	5.95	5.57	8.29	13.65	14.94	F = 7.92	0.001	η^2^ = 0.109
Diagnosable severity of ADHD symptomatology based on ADHD RS_TOT_ (N)	0	0.00	3	5.56	6	23.08	χ^2^ = 14.94	0.001	Cramer’s V = 0.335
Cardiology ICU stay (days)	n/a		6.01	4.955	5.53	4.87	t = 0.40	0.694	d = 0.096
Hospital stay (days)	n/a		19.87	12.34	15.58	6.28	t = 1.98	0.051	d = 0.438
Operation time (min)	n/a		207.86	86.41	203.80	82.14	t = 0.20	0.846	d = 0.048
ECC time (min)	n/a		106.94	51.89	103.80	70.11	t = 0.22	0.829	d = 0.005
Aortic cross-clamp time (min)	n/a		56.00	36.27	60.67	53.13	t = −0.40	0.692	d = −0.102
RACHS	n/a		2.78	0.90	2.77	1.37	t = 0.033	0.973	d = −0.008
Mechanical ventilation time (hours)	n/a		63.45	70.15	38.25	98.28	t = 1.26	0.212	d = 0.295
Number of operations	n/a		2.15	1.12	1.42	0.8	t = 3.293	0.002	d = 0.747
Cyanotic malformation (N)	n/a		37	68.51	6	23.07	χ^2^ = 14.58	< 0.001	Cramer’s V = 0.427

Mean/N: mean is reported for continuous and frequency for categorical variables

SD/%: standard deviation is reported for continuous and percentage for categorical variables

^a^ The comparisons involved all three groups regarding the sociodemographic and ADHD-related variables, while the two surgically treated subgroups only regarding the perioperative variables

*YSR*_*AD*_ Youth Self Report Attention Disorders Subscale, *CBCL*_*AD*_ Child Behaviour Checklist Attention Disorders Subscale, *ADHD RS*_*IA*_ ADHD Rating Scale–IV Inattention Subscale, *ADHD RS*_*HI*_ ADHD Rating Scale–IV Hyperactivity-Impulsivity Subscale, *ADHD RS*_*TOT*_ ADHD Rating Scale–IV Total Score, *ICU* intensive care unit, *ECC* extracorporeal circulation, *RACHS* Risk Assessment for Congenital Heart Surgery Score

The surgery group enrolled a convenience sample^1^ of parents and 97 school-aged patients with congenital heart disease who underwent their first cardiac surgery with extracorporeal circulation between 1995 and 2012 at the Paediatric Cardiac Centre of the Gottsegen György Hungarian Institute of Cardiology in Budapest. Exclusion criteria were prenatal injury (*n* = 2), cerebral palsy (*n* = 3), severe hypoxic-injury during peripartum periods (*n* = 2), genetic syndromes (Down or DiGeorge syndrome) associated with cognitive deficits (*n* = 2), lack of parental or children consent (*n* = 1), the incapability of completing questionnaires (*n* = 1), premature birth (*n* = 2), lack of perioperative data (*n* = 2), and missing ADHD questionnaire data (*n* = 2) – resulting in a sample size of 80. In the first subgroup of patients undergoing cardiac surgery, 54 children were enrolled if having the first surgery under 3 years of age, while the second subgroup included 26 patients who received the first operation at or above 3 years of age. Patients underwent 174 operations in total: 130 operations in the subgroup operated below the age of 3 for the first time and 44 operations in the other subgroup (diagnoses and surgical procedures can be found in a supplementary table, Table S1). The psychological examination was conducted in 2013 and 2014 during ambulatory check-ups, with an average of 10.5 ± 3.3 years (51% male) after the first surgery.

The control group included a convenience sample of 53 children (mean age at survey completion 11.79 ± 3.65 years, 45% male) who underwent an ambulatory investigation in 2013 and 2014 at the same center because of accidentally detected heart murmurs but later were identified as healthy. No study participants were born with extreme low weight (< 2500 g) or had known intrauterine growth retardation either in the control or the two intervention groups. In the control group, a single patient had a formal diagnosis of ADHD and treated by psychotherapy. In the surgery subgroups, no patients were formally diagnosed with ADHD. No participants of the study received pharmacotherapy for ADHD symptoms.

### Measures

The demographic and perioperative medical data were retrieved retrospectively from the institutional medical record. Medical variables included the length of the cardiac intensive care unit- (ICU) and overall hospital stay; length of the surgery; and length of extracorporeal circulation (ECC) needed while surgical interventions were made. Further, length of aortic cross-clamp use and the risk adjustment system for congenital heart surgery (RACHS) score were also reported. The former is a surgical instrument used during interventions without extracorporeal circulation, which separates the aorta from the outflow deriving from the heart excluding it from the systemic circulation, while the latter refers to the complexity of the operation (ranging from 0 to 6, where 6 represents the most complex form of surgery) [21].

The Hungarian version [22–24] of the Child Behaviour Checklist (CBCL; to be completed by parents), the Youth Self Report (YSR; to be completed by children) and the ADHD Rating Scale–IV (to be completed by parents) were used to assess ADHD symptomatology. The Child Behaviour Checklist (CBCL) and the Youth Self Report (YSR) measure a wide-range of emotional and behavioral problems of children [25]. The CBCL is to be completed by parents of children aged 6–18, while the YSR is to be completed by youth aged 11–18 themselves. Both scales are comprised of items measuring the same 6 domains, out of which the Attention Disorders Subscale (CBCL_AD_ and YSR_AD_) was used in the present study. Internal consistency for scores on the CBCL_AD_ was good (Cronbach’s alpha = 0.82), while that of scores on the YSR_AD_ was suboptimal but acceptable (Cronbach’s alpha = 0.63) in the present sample. In both cases, higher scores indicate more severe ADHD symptomatology. Diagnostic cut off scores for the CBCL_AD_ were used considering T scores of 65 (94th percentile) based on gender- and age specific norms [26]. Using this cut-off score, the prevalence of ADHD was estimated in our sample (Table 1). Even though this method is not equivalent to an expert diagnosis, these prevalence data (Table 1) can provide helpful information for the purpose of inter-study comparability of samples.

Beyond the total score (ADHD RS_TOT_), the 18-item ADHD Rating Scale–IV [27] provides two subscale scores pertaining to inattention (ADHD RS_IA_) and hyperactivity-impulsivity (ADHD RS_HI_) symptoms. This assessment tool is appropriate for use regarding children aged 4–20. In this study, the ‘home version’ of the instrument was used as parents were the raters (in contrast to the ‘school version’ where teachers report on the children), who characterized their child’s behavior considering the 6 months prior to assessment. Internal consistency in the present sample was very high both for the two subscales (Cronbach’s alpha = 0.95 in both cases) and the total (Cronbach’s alpha = 0.97) scores. Higher scores indicate more severe symptomatology in this case as well. To provide an approximate prevalence of ADHD in the sample (Table 1) according to this assessment tool, diagnostic cut off scores for the ADHD RS_TOT_ were used corresponding to T scores of 64.5 (93th percentile) based on gender- and age specific norms [28].

### Statistical analyses

Statistical analyses were performed using the SPSS 27.0 software (SPSS, Chicago, IL, USA). On the univariate level, chi square tests (for categorical variables), F-tests (for continuous variables where data were applicable for all three groups), and t-tests (for continuous variables where data were relevant for the two surgically treated groups only) were used to compare participants across surgery status (no cardiac surgery, first cardiac surgery under 3 years of age, first cardiac surgery at or above 3 years of age).

On the multivariate level, analysis of covariance (ANCOVA) was used to investigate the relationship between surgery status and the five indictors of ADHD symptom severity (YSR_AD_, CBCL_AD_, ADHD RS_IA_, ADHD RS_HI_, ADHD RS_TOT_), used as separate dependent variables. In addition to the omnibus tests, post-hoc pairwise comparisons were also performed to provide a more nuanced picture of the differences among the study groups. All multivariate analyses (including the post hoc tests) were controlled for sex and age at survey completion. To estimate statistical power of the multivariate models, post hoc power analysis was also carried for the main independent variable of interest (surgery status).

In case of both bivariate and multivariate analyses, estimates of effect size were also presented. We considered 0.2 as a threshold for small effect, 0.5 for moderate effect, and 0.8 for large effect in case of Cohen’s d; and 0.01 as a threshold for small effect, 0.06 for moderate effect, and 0.14 for large effect in case of η^2^ [29]; while the corresponding thresholds for Cramer’s V were 0.05, 0.10 and 0.15, respectively [30].

## Results

### Descriptive and univariate analyses

Demographic, perioperative and ADHD-related characteristics of the sample are described in Table 1. There was no statistically significant difference among the three study groups in terms of participants’ sex. However, those undergoing their first cardiac surgery later in life were older at survey completion (medium effect size) than members of the control group or who were operated at or below the age of 3 for the first time. Between the two surgically treated groups, there were statistically significant differences in the length of hospital stay (medium effect size), the number of operations (medium effect size), and the frequency of perioperative cyanosis (large effect size); in all three cases, values were higher (less favorable) for children who were first operated below 3 years of age (Table 1).

In terms of ADHD symptomatology, those undergoing surgery later in life had higher ADHD symptom scores across all five indicators than the other two subgroups; however, the difference did not reach statistical significance in case of the CBCL_AD_ (Table 1). The magnitude of the differences was small (CBCL_AD_) or moderate (YSR_AD_, ADHD RS_IA_, ADHD RS_HI_, ADHD RS_TOT_). In terms of the prevalence of diagnosable ADHD symptom severity, those undergoing surgery later in life had higher prevalence rates than the other two subgroups. The difference did not reach statistical significance in case of the CBCL_AD_ (although the effect size indicator suggested a moderate difference), while in the case of the ADHD RS_TOT_, the difference was statistically significant and the effect size was large (Table 1).

### Multivariate analyses

Results of the omnibus tests from the multivariate analyses indicated that surgery status was significantly associated with most indicators of ADHD symptomatology even after controlling for sex and age at survey completion, with effect sizes in the small to medium range (Table 2). Similar to the bivariate analyses, the only ADHD symptom severity indicator regarding which surgery status was not a statistically significant correlate was the CBCL_AD_ (Table 2). Observed (post hoc) power for the surgery status variable ranged from 0.43 (CBCL_AD_) to 0.75 (YSR_AD_).

**Table 2:** Relationship between ADHD symptomatology and surgery status in the multivariate analyses (ANCOVA)

	YSR_AD_ (*N* = 133)			CBCL_AD_ (*N* = 129)			ADHD RS_IA_ (*N* = 133)			ADHD RS_HI_ (*N* = 133)			ADHD RS_TOT_ (*N* = 133)		
	F	p	η^2^	F	p	η^2^	F	p	η^2^	F	p	η^2^	F	p	η^2^
Sex	0.308	0.580	0.002	0.001	0.979	< 0.001	0.298	0.586	0.002	0.017	0.897	< 0.001	0.184	0.669	0.001
Surgery status	4.416	0.014	0.065	2.107	0.126	0.033	3.951	0.022	0.058	3.038	0.051	0.045	4.308	0.015	0.063
Age at survey	0.021	0.885	< 0.001	0.096	0.757	0.001	0.010	0.920	< 0.001	3.579	0.061	0.027	1.134	0.289	0.009

*YSR*_*AD*_ Youth Self-Report Attention Disorders Subscale, *CBCL*_*AD*_ Child Behaviour Checklist Attention Disorders Subscale, *ADHD RS*_*IA*_ ADHD Rating Scale–IV Inattention Subscale, *ADHD RS*_*HI*_ ADHD Rating Scale–IV Hyperactivity-Impulsivity Subscale, *ADHD RS*_*TOT*_ ADHD Rating Scale–IV Total Score, *η*^*2*^ partial eta squared

Regarding all ADHD symptom severity indicators, the post hoc tests indicated that the symptoms of those treated surgically at or above 3 years of age were more severe than not only the control group but also of those who were treated surgically at a younger age; and with some exceptions, these differences were statistically significant (Table 3). Interestingly, the control group and those treated surgically under 3 years of age did not differ significantly across any of the five ADHD symptom severity indicators according to the post hoc tests.

**Table 3:** Results of the post-hoc tests regarding the relationship between surgery status and ADHD symptomatology (ANCOVA)

	YSR_AD_ (*N* = 133)		CBCL_AD_ (*N* = 129)		ADHD RS_IA_ (*N* = 133)		ADHD RS_HI_ (*N* = 133)		ADHD RS_TOT_ (*N* = 133)	
	EMM (SE)	Post hoc comparison	EMM (SE)	Post hoc comparison	EMM (SE)	Post hoc comparison	EMM (SE)	Post hoc comparison	EMM (SE)	comparison
Control (a)	2.487 (0.361)	p _a vs. b_ = 0.640 p _a vs. c_ = 0.004 p _b vs. c_ = 0.013	2.027 (0.412)	p _a vs. b_ = 0.433 p _a vs. c_ = 0.042 p _b vs. c_ = 0.160	2.812 (0.694)	p _a vs. b_ = 0.624 p _a vs. c_ = 0.018 p _b vs. c_ = 0.007	2.646 (0.552)	p _a vs. b_ = 0.293 p _a vs. c_ = 0.097 p _b vs. c_ = 0.015	5.608 (1.203)	p _a vs. b_ = 0.934 p _a vs. c_ = 0.008 p _b vs. c_ = 0.007
Surgically treated under 3 years of age (b)	2.725 (0.361)		2.490 (0.423)		2.332 (0.693)		1.825 (0.552)		5.467 (1.202)	
Surgically treated at or above 3 years of age (c)	4.389 (0.541)		3.581 (0.628)		5.821 (1.040)		4.327 (0.828)		11.495 (1.803)	

*YSR*_*AD*_ Youth Self Report Attention Disorders Subscale, *CBCL*_*AD*_ Child Behaviour Checklist Attention Disorders Subscale, *ADHD RS*_*IA*_ ADHD Rating Scale–IV Inattention Subscale, *ADHD RS*_*HI*_ ADHD Rating Scale–IV Hyperactivity-Impulsivity Subscale, *ADHD RS*_*TOT*_ ADHD Rating Scale–IV Total Score, *EMM (SE)* estimated marginal means adjusted for sex and age at survey completion (standard error)

### Sensitivity analyses

Considering the non-trivial differences in standard deviation of some ADHD indicators across study groups (Table 1), the multivariate analyses were also rerun on a trimmed data set eliminating outliers, using the criterion of a z score > |3.29|. In this case, surgery status was only associated with YSR_AD_ scores (F = 3.126, *p* = 0.047, η^2^ = 0.047) but not with the other four ADHD symptomatology indicators (CBCL_AD_: F = 1.093, *p* = 0.339, η^2^ = 0.017; ADHD RS_IA_: F = 0.777, *p* = 0.462, η^2^ = 0.012; ADHD RS_HI_: F = 1.887, *p* = 0.156, η^2^ = 0.029; ADHD RS_TOT_: F = 1.720, *p* = 0.183, η^2^ = 0.027).

Given the importance of sex differences in the phenomenology of ADHD, further multivariate analyses were completed (in the untrimmed data set) to test the moderator role of sex: an interaction term of sex and surgery status was added to the default models. This interaction term was significant in the model for the YSR_AD_ (*p* = 0.031) and the CBCL_AD_ (*p* = 0.036); while non-significant for the model for ADHD RS_IA_ (*p* = 0.073), ADHD RS_HI_ (*p* = 0.355), and the ADHD RS_TOT_ (*p* = 0.284). Considering the significant interactions, sex-stratified analyses were also conducted for scores on the YSR_AD_ and the CBCL_AD_. These analyses indicated that surgery status was significantly associated with YSR_AD_ (F = 6.248, *p* = 0.003, η^2^ = 0.159) and CBCL_AD_ (F = 4.441, *p* = 0.016, η^2^ = 0.122) scores in girls but not in boys (YSR_AD_: F = 0.659, *p* = 0.521, η^2^ = 0.022; CBCL_AD_: F = 1.040, *p* = 0.360, η^2^ = 0.035).

## Discussion

### Interpretation of the results

The worldwide prevalence of ADHD in pediatric populations is approximately 5.29% [31]. ADHD is often co-morbid with behavioral disorders (36–40%), major depressive episodes (18%), anxiety problems (26%) [32] and learning disabilities (30%) [33]. Quality of life of affected patients is lower, even if in half of the cases, ADHD symptoms disappear upon reaching adulthood [8, 34].

The aim of this study was to shed light on the long-term consequences of pediatric cardiac surgery in terms of attention-deficit and hyperactivity symptoms. To the best of our knowledge, this is the first study reporting an age-related comparison of patients with congenital heart disease undergoing cardiac surgery and a healthy population. The results indicated a significantly higher severity of attention deficit and hyperactivity symptoms in children who underwent cardiac surgery at or above the age of three compared to non-operated individuals or even children operated at a younger age, an outcome which was largely consistent across assessment tools and data providers (patient- vs. parent-report). Interestingly, the subgroup operated at a younger age underwent a *larger* number of operations and was *more* frequently diagnosed with a cyanotic malformation, which suggests that factors other than cyanotic malformation or number of operations (cf. repeated anesthetic exposure) should explain the more severe ADHD symptomatology observed in the subgroup operated later in life.

A potential explanation for our findings is that repair mechanisms and neuronal plasticity in newborns and younger children are more efficient than in children of older ages. Brain development consists of a cascade of different stages composing of cell birth, cell migration, differentiation, maturation (dendrite and axon growth), synaptogenesis, cell death, synaptic pruning, and myelogenesis – all following an age-dependent pattern [20]. Due to the uncertainty in the number of neurons reaching their predicted and appropriate destination, the brain overproduces both neurons and synapses with a peak of formation occurring between the ages of one and 2 years [20, 35]. During this stage, unneeded or damaged neurons are removed by synaptic pruning and cell death, while stem cells lining the subventricular zone are activated and parallel systems initiate to form new cell connections. Additionally, dendrites and spines show particularly high plasticity in response, forming synapses in just a few hours after injury [20]. Due to these facts, developmental outcomes from different injuries will vary according to the exact stage of development [35, 36] with individuals below the age of three having better chances for full recovery. Further research is needed to further confirm these explorative findings suggesting age-dependent behavioral outcomes in relation to pediatric cardiac surgery.

It is also worthy of note though that the results of the primary analyses of this study were not particularly robust: the sensitivity analyses indicated that the trends observed in the primary analyses emerge more consistently among females than males and that outliers might be responsible for a disproportionately large amount of variance. These considerations suggest that further investigations with a more explicit focus on sex differences and larger samples need to confirm the findings of this exploratory study and investigate potential moderators of the relationship between age at cardiac surgery and ADHD symptomatology.

### Strengths and limitations

A major strength of this study was the consideration of both self- and collateral reports on the same construct (ADHD symptomatology) using multiple, parallel assessment tools. While overall the findings were homogeneous, certain differences did emerge across assessment tools (e.g., difference in effect sizes, prevalence of diagnosable severity of ADHD symptomatology), which indicates that future studies with a similar focus should carefully select which measure(s) to use considering, for instance, the goals of the study (e.g., focus on inattention versus the full range of ADHD symptoms) or the statistical power intended to achieve.

Limitations of this study need to be acknowledged as well. First, the sample was relatively small limiting our ability to reliably detect small differences among the study groups or conduct adequately powered sex-stratified analyses. This shortcoming of the study was also highlighted by the suboptimally low (< 0.80) post hoc power values. Further, the convenience nature of the sampling procedure limits the generalizability of the findings and reduces the strength of our hypothesis regarding neural plasticity. For example, it is possible that the subsample of those treated surgically at a younger age had a more favorable ADHD symptom profile in our study compared to those operated on later not because of more effective neural repair mechanisms but because only the most resilient individuals from the first group survived their early years (cf. the higher number and complexity of cardiac operations and more frequent cyanotic malformation in this group suggesting a higher risk of death) raising the possibility of selection bias.^2^

Another sampling-related limitation of the study design was the heterogeneity of the sample, that is, congenital heart disease was conceptualized as a homogenous disease despite the quite diverse conditions this umbrella term covers. The large variability in medical procedures used to correct this diverse group of medical conditions makes it challenging to design studies with adequate sample size while focusing on a single condition or surgical procedure. Most likely not independently of this fact, the omnibus approach used in the present study is not unique and was also used in other studies investigating the psychological aspects of congenital heart diseases or their treatment [37, 38].

Yet another limitation of the present study is that certain surgical interventions – from surgical technology to treatment guidelines for surgery and postoperative clinical management – have evolved considerably since the study period; and therefore, our findings might be less generalizable to clients receiving treatment according to these more recent protocols. Further studies following the most recent treatment guidelines and focusing on a single or smaller group of congenital heart diseases and their treatment could contribute to a more nuanced understanding of how congenital heart diseases and the surgical procedures aiming to correct them influence neurocognitive development.

A further limitation of the study was the lack of access to detailed medical information on the prenatal and the peripartum periods (e.g., intrauterine growth retardation, smoking behavior during pregnancy and other teratogenic factors), which could also have influenced the cognitive and behavioral development of the offspring [39–41]. Data on families’ socio-economic status, life style factors, or parents’ psychological characteristics were not collected either, all of which may also contribute to the development, prognosis, or reporting of ADHD symptoms [42, 43]. In addition, the present study did not include any detailed genetic analyses either, thus the overlap with non-cardiac congenital conditions presenting only later in life (post-surgery) also remained unknown. Considering these variables in future research could increase the reliability of findings in studies with a similar focus. Finally, due to the methodology of the current study, we cannot rule out the possibility that the reported ADHD symptoms could have been better explained by mental health conditions other than ADHD (e.g., anxiety, depression); using diagnostic evaluations by mental health experts could have contributed to a more reliable and valid ADHD assessment protocol.

### Clinical implications and conclusions

The results of the present study raise the possibility that the age of children at cardiac surgery has a prognostic role in the emergence of a future attention disorder. The clinical relevance of routine ADHD screening and early diagnosis in pediatric cardiac rehabilitation could help both patients and their social environment to recognize early signs and prevent severe consequences of ADHD such as poorer school performance, social isolation, depression, or substance abuse. Our study also calls attention to the importance of the large variety of factors potentially influencing behavioral outcomes in pediatric cardiac populations (number of surgeries, possible surgical complications, type of malformations etc.), which variables should be made easily accessible to health care providers in (electronic) health records to facilitate both high-quality clinical service delivery and research.

The results of the present study suggest that the negative psychological effects of pediatric cardiac surgery might not be equally salient in different ages and thus certain age groups might be more at risk to develop ADHD symptoms after cardiac surgery. This notion, on the one hand, could help better target ADHD screening, assessment, and treatment procedures in pediatric populations, while on the other hand, it raises the possibility that other cardiac-surgery-related psychological outcomes are also dependent on the timing of cardiac interventions, which possibility deserves further investigations.

## Supplementary Information

**Additional file 1: Table S1.** Diagnoses and surgical procedures in the sample, stratified by age at first operation (below vs. at or above 3 years of age).

## Data Availability

The raw data of the present study may not be shared publicly because the data set contains sensitive personal health information which - considering the relatively low number of participants and the very specific setting - could jeopardize participants’ privacy rights. Therefore, data are available for researchers only who meet the criteria for access to confidential data. Please address any such requests to Andrea Székely, M.D., Ph.D., szekelya@kardio.hu.

## Footnotes

1 Enrollment was most significantly influenced by two factors, namely 1) likelihood of children showing up for check-up appointments (parents whose children are doing well often do not bring their children for check-ups a decade after surgery) and 2) match with the research staff’s hours of availability.

2 We estimate though that the likelihood of such selection bias is relatively low. The average operation-related annual mortality rate in the given cardiac center during the study period was 4.24% [37]. Even if assuming that *all* patients who died, had they survived, would have been part of the subgroup of clients operated under the age of 3 and *all* would have been characterized by a diagnosable severity level of ADHD symptomatology, that would increase the number of ADHD diagnoses in this study group from 3 (5.88%) to 5 (9.26%). Even in this case, the estimated ADHD prevalence rates in the subgroup operated above at or above 3 years of age remained higher (12–23%, depending on assessment tool, cf. Table 1).

## References

[1] Khairy P, Ionescu-Ittu R, Mackie AS, Abrahamowicz M, Pilote L, Marelli AJ (2010). Changing mortality in congenital heart disease. J Am Coll Cardiol.

[2] Marino BS, Lipkin PH, Newburger JW, Peacock G, Gerdes M, Gaynor JW, Mussatto KA, Uzark K, Goldberg CS, Johnson WH (2012). Neurodevelopmental outcomes in children with congenital heart disease: evaluation and management: a scientific statement from the American Heart Association. Circulation.

[3] Bellinger DC, Wypij D, Kuban KC, Rappaport LA, Hickey PR, Wernovsky G, Jonas RA, Newburger JW (1999). Developmental and neurological status of children at 4 years of age after heart surgery with hypothermic circulatory arrest or low-flow cardiopulmonary bypass. Circulation.

[4] Holst LM, Kronborg JB, Jepsen JRM, Christensen JØ, Vejlstrup NG, Juul K, Bjerre JV, Bilenberg N, Ravn HB (2020). Attention-deficit/hyperactivity disorder symptoms in children with surgically corrected ventricular septal defect, transposition of the great arteries, and tetralogy of Fallot. Cardiol Young.

[5] Sistino JJ, Atz AM, Ellis C, Simpson KN, Ikonomidis JS, Bradley SM (2015). Association between method of cerebral protection during neonatal aortic arch surgery and attention deficit/hyperactivity disorder. Ann Thorac Surg.

[6] Sistino JJ, Atz AM, Simpson KN, Ellis C, Ikonomidis JS, Bradley SM (2015). The prevalence of attention-deficit/hyperactivity disorder following neonatal aortic arch repair. Cardiol Young.

[7] Shillingford AJ, Glanzman MM, Ittenbach RF, Clancy RR, Gaynor JW, Wernovsky G (2008). Inattention, hyperactivity, and school performance in a population of school-age children with complex congenital heart disease. Pediatrics.

[8] DeMaso DR, Calderon J, Taylor GA, Holland JE, Stopp C, White MT, Bellinger DC, Rivkin MJ, Wypij D, Newburger JW (2017). Psychiatric disorders in adolescents with single ventricle congenital heart disease. Pediatrics.

[9] Hovels-Gurich HH, Konrad K, Skorzenski D, Herpertz-Dahlmann B, Messmer BJ, Seghaye MC (2007). Attentional dysfunction in children after corrective cardiac surgery in infancy. Ann Thorac Surg.

[10] Yamada DC, Porter AA, Conway JL, LeBlanc JC, Shea SE, Hancock-Friesen CL, Warren AE (2013). Early repair of congenital heart disease associated with increased rate of attention deficit hyperactivity disorder symptoms. Can J Cardiol.

[11] Sarrechia I, Miatton M, Francois K, Gewillig M, Meyns B, Vingerhoets G, De Wolf D (2015). Neurodevelopmental outcome after surgery for acyanotic congenital heart disease. Res Dev Disabil.

[12] Quartermain MD, Ittenbach RF, Flynn TB, Gaynor JW, Zhang X, Licht DJ, Ichord RN, Nance ML, Wernovsky G (2010). Neuropsychological status in children after repair of acyanotic congenital heart disease. Pediatrics.

[13] DeMaso DR, Beardslee WR, Silbert AR, Fyler DC (1990). Psychological functioning in children with cyanotic heart defects. J Dev Behav Pediatr.

[14] Sprung J, Flick RP, Katusic SK, Colligan RC, Barbaresi WJ, Bojanić K, Welch TL, Olson MD, Hanson AC, Schroeder DR, Wilder RT, Warner DO (2012). Attention-deficit/hyperactivity disorder after early exposure to procedures requiring general anesthesia. Mayo Clin Proc.

[15] Xu L, Hu Y, Huang L, Liu Y, Wang B, Xie L, Hu Z (2019). The association between attention deficit hyperactivity disorder and general anaesthesia - a narrative review. Anaesthesia.

[16] Flick RP, Katusic SK, Colligan RC, Wilder RT, Voigt RG, Olson MD, Sprung J, Weaver AL, Schroeder DR, Warner DO (2011). Cognitive and behavioral outcomes after early exposure to anesthesia and surgery. Pediatrics.

[17] Holtby HM (2012). Neurological injury and anesthetic neurotoxicity following neonatal cardiac surgery: does the head rule the heart or the heart rule the head?. Futur Cardiol.

[18] Gaynor JW, Wernovsky G, Jarvik GP, Bernbaum J, Gerdes M, Zackai E, Nord AS, Clancy RR, Nicolson SC, Spray TL (2007). Patient characteristics are important determinants of neurodevelopmental outcome at one year of age after neonatal and infant cardiac surgery. J Thorac Cardiovasc Surg.

[19] Fuller S, Nord AS, Gerdes M, Wernovsky G, Jarvik GP, Bernbaum J, Zackai E, Gaynor JW (2009). Predictors of impaired neurodevelopmental outcomes at one year of age after infant cardiac surgery. Eur J Cardiothorac Surg.

[20] Kolb B, Gibb R (2011). Brain plasticity and behaviour in the developing brain. J Can Acad Child Adolesc Psychiatry.

[21] Simsic JM, Cuadrado A, Kirshbom PM, Kanter KR (2006). Risk adjustment for congenital heart surgery (RACHS): is it useful in a single-center series of newborns as a predictor of outcome in a high-risk population?. Congenit Heart Dis.

[22] Gádoros J (1996). Szociodemográfiai rizikótényezők vizsgálata gyermek viselkedési kérdőív alkalmazásával. Psychiat Hung.

[23] Rózsa S, Gádoros J, Kő N (1999). A gyermekpszichiátriai zavarok kérdőíves mérése: A gyermekviselkedési kérdőív diagnosztikai megbízhatósága és a több információforráson alapuló jellemzések egyezése. Psychiatr Hung.

[24] Perczel-Forintos D, Kiss Z, Ajtay G, Perczel-Forintos D, Kiss Z, Ajtay G (2005). Hiperkinetikus Zavar Kérdőív. Kérdőívek, becslőskálák a klinikai pszichológiában.

[25] Achenbach TM, Ruffle TM (2000). The child behavior checklist and related forms for assessing behavioral/emotional problems and competencies. Pediatr Rev.

[26] Rózsa S, Kő N, Gádoros J (2015). A Gyermekviselkedési Kérdőív.

[27] DuPaul G, Power T, Anastopulos A, Reid R (1998). ADHD rating scale IV: checklist, norms, and clinical interpretation.

[28] Dobrean A, Păsărelu C-R, Balazsi R, Predescu E: Measurement invariance of the ADHD rating scale–IV home and school versions across age, gender, clinical status, and informant. Assessment in press. 10.1177/1073191119858421 31253044

[29] Cohen J (1992). A power primer. Psychol Bull.

[30] Akoglu H (2018). User's guide to correlation coefficients. Turk J Emerg Med.

[31] Polanczyk G, De Lima MS, Horta BL, Biederman J, Rohde LA (2007). The worldwide prevalence of ADHD: a systematic review and metaregression analysis. Am J Psychiatry.

[32] Green M, Wong M, Atkins D, Taylor J, Feinleib M (1999). Diagnosis of attention-deficit/hyperactivity disorder.

[33] Wolraich ML, Wibbelsman CJ, Brown TE, Evans SW, Gotlieb EM, Knight JR, Ross EC, Shubiner HH, Wender EH, Wilens T (2005). Attention-deficit/hyperactivity disorder among adolescents: a review of the diagnosis, treatment, and clinical implications. Pediatrics.

[34] Kasmi L, Bonnet D, Montreuil M, Kalfa D, Geronikola N, Bellinger DC, et al. Neuropsychological and psychiatric outcomes in dextro-transposition of the great arteries across the lifespan: A state-of-the-art review. Front Pediatr. 2017;5:59. 10.3389/fped.2017.00059 PMC5364136 28393063

[35] Kolb B, Gibb R, Gorny G (2003). Experience-dependent changes in dendritic arbor and spine density in neocortex vary qualitatively with age and sex. Neurobiol Learn Mem.

[36] Gregg CT, Shingo T, Weiss S (2001). Neural stem cells of the mammalian forebrain. Symp Soc Exp Biol.

[37] Menahem S, Poulakis Z, Prior M (2008). Children subjected to cardiac surgery for congenital heart disease. Part 1 - emotional and psychological outcomes. Interact Cardiovasc Thorac Surg.

[38] Pike NA, Woo MA, Poulsen MK, Evangelista W, Faire D, Halnon NJ, et al. Predictors of Memory Deficits in Adolescents and Young Adults with Congenital Heart Disease Compared to Healthy Controls. Front Pediatr. 2016;4:117. 10.3389/fped.2016.00117 PMC5086579 27843890

[39] Knopik VS (2009). Maternal smoking during pregnancy and child outcomes: real or spurious effect?. Dev Neuropsychol.

[40] Roy TS, Seidler FJ, Slotkin TA (2002). Prenatal nicotine exposure evokes alterations of cell structure in hippocampus and somatosensory cortex. J Pharmacol Exp Ther.

[41] Ramsay MC, Reynolds CR (2000). Does smoking by pregnant women influence IQ, birth weight, and developmental disabilities in their infants? A methodological review and multivariate analysis. Neuropsychol Rev.

[42] Russell AE, Ford T, Russell G (2015). Socioeconomic associations with ADHD: findings from a mediation analysis. PLoS One.

[43] Rowland AS, Skipper BJ, Rabiner DL, Qeadan F, Campbell RA, Naftel AJ, Umbach DM (2018). Attention-deficit/hyperactivity disorder (ADHD): interaction between socioeconomic status and parental history of ADHD determines prevalence. J Child Psychol Psychiatry.

