# Supplementary material for: ADHD symptomatology of children with congenital heart disease 10 years after cardiac surgery: the role of age at operation
Source: BMC Psychiatry. 2021 Jun 24;21:316. doi: 10.1186/s12888-021-03324-w (PMC8223303; doi:10.1186/s12888-021-03324-w)
Supplement: Supplementary file 1 — Additional file 1: Table S1. Diagnoses and surgical procedures in the sample, stratified by age at first operation (below vs. at or above 3 years of age). [file 12888_2021_3324_MOESM1_ESM.docx]

**ADHD symptomatology of children with congenital heart disease 10 years after cardiac surgery: The role of age at operation**

Nikoletta R. Czobor, Zsófia Ocsovszky, György Roth, Szabolcs Takács, Márta Csabai, Edgár Székely, János Gál, Andrea Székely, &

Barna Konkolÿ Thege

**Table S1. Diagnoses and surgical procedures in the sample, stratified by age at first operation (below vs. above 3 years of age). Please see list of abbreviations at the end of the file.**

| **Operated <3-years (n=54)** | | | | | | | | | | | | | | | | |
| --- | --- | --- | --- | --- | --- | --- | --- | --- | --- | --- | --- | --- | --- | --- | --- | --- |
| **Diagnosis-1** | | **Diagnosis-2** | **Diagnosis-3** | **Procedure date-1** | **Age (months)** | **Procedure-1** | **Procedure date-2** | **Age (years)** | **Procedure-2** | **Procedure date-3** | **Age (years)** | **Procedure-3** | **Procedure date-4** | **Age (years)** | **Procedure-4** | **Procedure-5 (if any) or other procedures** |
| 1 | Aortic coarctation | Mitral stenosis |  | 2004 | 3 | EEEA | 2006 | 2 | MVR | 2017 | 13 | MVR | 2017 | 13 | OPCABG |  |
| 2 | Aortic coarctation | Aortic valve stenosis |  | 2004 | 5 | EEEA | 2018 | 14 | AVR |  |  |  |  |  |  |  |
| 3 | ASD | DCM |  | 2005 | <1 | ASD closure | 2010 | 5 | Berlinheart LVAD | 2010 | 5 | HTX |  |  |  | Non-CPB pacemaker operations, Vena Cava Inferior stenting |
| 4 | CAVD |  |  | 2005 | <1 | Repair (two-patch) | 2005 | <1 | MVR | 2012 | 7 | MVR, tricuspidal valve plasty |  |  |  |  |
| 5 | CAVD |  |  | 2006 | <1 | Repair (two patch) |  |  |  |  |  |  |  |  |  |  |
| 6 | DORV | VSD |  | 2004 | <1 | VSD closure |  |  |  |  |  |  |  |  |  |  |
| 7 | DORV | Pulmonary valve stenosis |  | 1995 | 2 | DORV repair with RV obstruction |  |  |  |  |  |  |  |  |  | Non-CPB pacemaker operations |
| 8 | DORV | Malposition of great arteries |  | 1995 | <1 | mBT-shunt | 1999 | 4 | Glenn-shunt | 2003 | 7 | TCPC, extracardiac, fenestrated | 2009 | 13 | shunt closure | Rashkind, fenestration closure attempt (2010) |
| 9 | HLHS | Aortic valve stenosis |  | 2004 | <1 | Norwood | 2005 | 1 | Glenn-shunt | 2008 | 4 | TCPC, extracardiac, nonfenestrated |  |  |  | BVP |
| 10 | HRHS | PA |  | 2002 | 10 | Systemic-to-pulmonary shunt | 2004 | 2 | Glenn-shunt | 2007 | 5 | TCPC, extracardiac, fenestrated |  |  |  | Fenestration closure |
| 11 | HRHS |  |  | 1997 | <1 | Systemic-to-pulmonary shunt | 1998 | 1 | Glenn-shunt | 2001 | 4 | TCPC, extracardiac, fenestrated |  |  |  |  |
| 12 | HRHS | Pulmonary valve stenosis |  | 2001 | <1 | Systemic-to-pulmonary shunt | 2002 | 1 | Systemic-to-pulmonary shunt, transannular patch, ASD augmentation | 2003 | 2 | Glenn-shunt | 2005 | 4 | extracardiac TCPC, fenestrated | BVP, fenestration closure |
| 13 | IAVD |  |  | 2000 | 15 | Repair |  |  |  |  |  |  |  |  |  |  |
| 14 | IAVD | Mitral valve stenosis |  | 2001 | 26 | Repair | 2002 | 1 | MVR | 2014 | 13 | MVR |  |  |  |  |
| 15 | Interrupted aortic arch | VSD |  | 2001 | <1 | Repair (patch), VSD closure | 2016 | 15 | AVR |  |  |  |  |  |  | BVP + Botall stent, reBVP |
| 16 | PA | VSD |  | 2000 | 3 | Systemic-to-pulmonary shunt | 2001 | 1 | Unifocalization (homograft) | 2016 | 16 | Pulmonary homograft |  |  |  |  |
| 17 | PA | VSD |  | 1998 | <1 | Systemic-to-pulmonary shunt | 2000 | 2 | Repair (shunt closure, VSD closure, pumonary homograft |  |  |  |  |  |  |  |
| 18 | PA | VSD |  | 2004 | <1 | Systemic-to-pulmonary shunt | 2005 | 1 | Repair (transannular patch) | 2017 | 13 | PVR (biological) |  |  |  |  |
| 19 | PA | VSD |  | 2001 | 7 | Systemic-to-pulmonary shunt | 2003 | 2 | VSD closure, pulmonary homograft |  |  |  |  |  |  | Resection of chest wall granuloma |
| 20 | PA | Ebstein-anomaly |  | 1997 | <1 | Systemic-to-pulmonary shunt | 1998 | 1 | Glenn-shunt | 2001 | 4 | TCPC, extracardiac, fenestrated |  |  |  | Fenestration closure |
| 21 | PAPVR | AV block |  | 2007 | <1 | Repair |  |  |  |  |  |  |  |  |  | Non-CPB pacemaker operations |
| 22 | PDA | Aortic valve stenosis |  | 2012 | 32 | AVR (biological) |  |  |  |  |  |  |  |  |  | PDA coil |
| 23 | Pulmonary atresia | VSD |  | 2004 | <1 | Systemic-to-pulmonary shunt | 2005 | 1 | Repair (VSD closure, pulmonary homograft) | 2012 | 8 | Pulmonary homograft | 2014 | 10 | Homograft | Stenting |
| 24 | Pulmonary valve stenosis |  |  | 2004 | <1 | Pulmonary valve extraction, transannular patch |  |  |  |  |  |  |  |  |  | BVP + PDA coil |
| 25 | Supravalvar pulmonary stenosis |  |  | 2004 | 10 | Repair (patch) |  |  |  |  |  |  |  |  |  |  |
| 26 | TA | VSD |  | 2001 | <1 | Systemic-to-pulmonary shunt | 2003 | 2 | Glenn-shunt | 2004 | 3 | TCPC, extracardiac, fenestrated |  |  |  | Rashkind |
| 27 | TA | VSD |  | 2003 | <1 | Pulmonary banding | 2003 | <1 | Systemic-to-pulmonary shunt, pulmonary ligation | 2003 | <1 | Unligation, pulmonary banding | 2005 | 2 | Glenn-shunt | Rashkind (2006); TCPC, extracardiac, fenestrated (2007) |
| 28 | Tetralogy of Fallot |  |  | 2008 | 2 | Repair |  |  |  |  |  |  |  |  |  |  |
| 29 | Tetralogy of Fallot | Supravalvar pulmonary stenosis |  | 2002 | 7 | Repair (transannular patch) | 2017 | 15 | PVR (biological) |  |  |  |  |  |  |  |
| 30 | Tetralogy of Fallot |  |  | 2003 | <1 | Repair (transannular patch) |  |  |  |  |  |  |  |  |  |  |
| 31 | Tetralogy of Fallot |  |  | 2005 | 11 | Repair |  |  |  |  |  |  |  |  |  |  |
| 32 | Tetralogy of Fallot |  |  | 2000 | 24 | Repair | 2003 | 3 | Pulmonary homograft | 2011 | 11 | Homograft reimplantation | 2014 | 14 | TVR (biological), PVR (biological) |  |
| 33 | TGA | VSD |  | 2007 | <1 | Systemic-to-pulmonary shunt | 2010 | 3 | Arterial switch |  |  |  |  |  |  | Rashkind |
| 34 | TGA | VSD |  | 2002 | <1 | Arterial switch |  |  |  |  |  |  |  |  |  |  |
| 35 | TGA |  |  | 2000 | 2 | Arterial switch |  |  |  |  |  |  |  |  |  |  |
| 36 | TGA | VSD |  | 2003 | <1 | Arterial switch |  |  |  |  |  |  |  |  |  | Non-CPB pacemaker operations |
| 37 | TGA | Ebstein-anomaly |  | 2003 | <1 | Subpulmonary resection, pulmonary plasty |  |  |  |  |  |  |  |  |  | Non-CPB pacemaker operations |
| 38 | TGA |  |  | 2001 | <1 | Arterial switch | 2013 | 12 | AVR, bilateral outflow tract resections |  |  |  |  |  |  |  |
| 39 | TGA |  |  | 2001 | 3 | Arterial switch |  |  |  |  |  |  |  |  |  | Rashkind |
| 40 | TGA | PA |  | 2003 | <1 | Systemic-to-pulmonary shunt | 2004 | 1 | Shunt, transannular patch | 2004 | 1 | Shunt | 2005 | 2 | Glenn-shunt | Stenting; TCPC, extracardiac, fenestrated (2007) |
| 41 | TGA |  |  | 2004 | <1 | Arterial switch |  |  |  |  |  |  |  |  |  | RFA |
| 42 | TGA | Aortic valve stenosis |  | 2004 | <1 | Systemic-to-pulmonary shunt | 2005 | 1 | Glenn-shunt | 2009 | 5 | TCPC, extracardiac, nonfenestrated |  |  |  | Rashkind (2010) |
| 43 | TGA | Hypoplastic aortic arch | VSD | 2000 | <1 | EEEA, pulmonary banding | 2003 | 3 | DKS, Glenn-shunt | 2005 | 5 | TCPC, extracardiac, nonfenestrated |  |  |  |  |
| 44 | TGA | HRHS | VSD | 2000 | 5 | TCPC, extracardiac, non-fenestrated |  |  |  |  |  |  |  |  |  | Non-CPB pacemaker operations |
| 45 | UVH |  |  | 2003 | <1 | Glenn-shunt | 2007 | 4 | TCPC, extracardiac, fenestrated |  |  |  |  |  |  | Fenestration closure |
| 46 | UVH | Malposition of the great arteries |  | 2000 | <1 | Pulmonary banding | 2001 | 1 | Systemic-to-pulmonary shunt |  |  |  |  |  |  |  |
| 47 | UVH | Malposition of the great arteries |  | 2002 | <1 | Systemic-to-pulmonary shunt | 2003 | 1 | Glenn-shunt | 2006 | 4 | TCPC, extracardiac, fenestrated |  |  |  | Fenestration closure |
| 48 | UVH | Malposition of the great arteries |  | 1999 | <1 | Systemic-to-pulmonary shunt | 2000 | 1 | Shunt, transannular patch | 2003 | 4 | Glenn-shunt | 2004 | 5 | TCPC, extracardiac, fenestrated | fenestration closure |
| 49 | UVH | Malposition of the great arteries |  | 2002 | 32 | TCPC, extracardiac, fenestrated |  |  |  |  |  |  |  |  |  |  |
| 50 | UVH |  |  | 2002 | 3 | Glenn-shunt | 2006 | 7 | TCPC, extracardiac, non-fenestrated |  |  |  |  |  |  |  |
| 51 | UVH | Malposition of great arteries |  | 2002 | 3 | Glenn-shunt | 2007 | 8 | TCPC, extracardiac, fenestrated |  |  |  |  |  |  | Fenestration closure |
| 52 | VSD |  |  | 2002 | 14 | VSD closure |  |  |  |  |  |  |  |  |  |  |
| 53 | VSD |  |  | 2004 | 23 | VSD closure |  |  |  |  |  |  |  |  |  |  |
| 54 | VSD |  |  | 2002 | <1 | VSD closure |  |  |  |  |  |  |  |  |  |  |

| **Operated >3-years (n=26)** | | | | | | | | | | | | | | | | |
| --- | --- | --- | --- | --- | --- | --- | --- | --- | --- | --- | --- | --- | --- | --- | --- | --- |
| **Diagnosis-1** | | **Diagnosis-2** | **Diagnosis-3** | **Procedure date-1** | **Age (years)** | **Procedure-1** | **Procedure date-2** | **Age (years)** | **Procedure-2** | **Procedure date-3** | **Age (years)** | **Procedure-3** | **Procedure date-4** | **Age (years)** | **Procedure-4** | **Procedure-5 (if any) or other procedures** |
| 1 | Aortic coarctation |  |  | 2011 | 4 | EEEA |  |  |  |  |  |  |  |  |  |  |
| 2 | Aortic valve regurgitation |  |  | 2008 | 6 | Ross |  |  |  |  |  |  |  |  |  |  |
| 3 | Aortic valve stenosis |  |  | 2008 | 5 | Ross |  |  |  |  |  |  |  |  |  | BVP |
| 4 | *Aortic valve stenosis | Aortic coarctation |  | 2006 | 10 | Ross | 2011 | 15 | Homografting (aortic) | 2015 | 19 | PVR (biological) |  |  |  | BVP |
| 5 | Aortic valve stenosis |  |  | 2009 | 8 | AVR |  |  |  |  |  |  |  |  |  | BVP |
| 6 | Aortic valve stenosis |  |  | 2002 | 6 | Ross |  |  |  |  |  |  |  |  |  | BVP |
| 7 | Aortic valve stenosis |  |  | 2011 | 12 | AVR |  |  |  |  |  |  |  |  |  | BVP |
| 8 | Aortic valve stenosis | Endocarditis |  | 2002 | 3 | AVR (homograft) | 2007 | 8 | AVR |  |  |  |  |  |  |  |
| 9 | Aortic valve stenosis |  |  | 2000 | 5 | Aortic plasty | 2001 | 6 | Ross | 2008 | 13 | Homograft | 2016 | 21 | Homograft reimplantation |  |
| 10 | ASD |  |  | 2004 | 7 | ASD closure |  |  |  |  |  |  |  |  |  |  |
| 11 | ASD, PSVT | Pacemaker induced cardiomyopathy |  | 2000 | 5 | ASD closure |  |  | Waiting for transplantation* |  |  |  |  |  |  | RFA (2000) |
| 12 | DCM |  |  | 2012 | 9 | HTX |  |  |  |  |  |  |  |  |  | Biopsies |
| 13 | DORV |  |  | 2005 | 9 | Repair (without RV obstruction) |  |  |  |  |  |  |  |  |  |  |
| 14 | Pulmonary valve stenosis |  |  | 2012 | 7 | PVR (biological) |  |  |  |  |  |  |  |  |  | BVP |
| 15 | *Pulmonary valve stenosis |  |  | 1998 | 3 | Systemic-to-pulmonary shunt | 2000 | 5 | Pulmonary plasty | 2008 | 13 | Pulmonary re-plasty |  |  |  | Rashkind, stentimplantation |
| 16 | *Tetralogy of Fallot |  |  | 1997 | 4 | Repair (transannular patch) | 2012 | 19 | PVR (biological) |  |  |  |  |  |  |  |
| 17 | *Tetralogy of Fallot |  |  | 2004 | 6 | Repair | 2013 | 15 | PVR (biological) |  |  |  |  |  |  |  |
| 18 | *TGA | HRHS |  | 2000 | 5 | TCPC, extracardiac, non-fenestrated |  |  |  |  |  |  |  |  |  | Non-CPB pacemaker operations |
| 19 | VSD |  |  | 2005 | 5 | VSD closure |  |  |  |  |  |  |  |  |  |  |
| 20 | VSD |  |  | 2010 | 6 | VSD closure |  |  |  |  |  |  |  |  |  |  |
| 21 | VSD |  |  | 2009 | 9 | VSD closure |  |  |  |  |  |  |  |  |  |  |
| 22 | VSD |  |  | 2011 | 7 | VSD closure |  |  |  |  |  |  |  |  |  | Abdominal surgery |
| 23 | VSD |  |  | 2012 | 10 | VSD closure |  |  |  |  |  |  |  |  |  |  |
| 24 | VSD |  |  | 2005 | 8 | VSD closure |  |  |  |  |  |  |  |  |  | Abdominal, facial operations |
| 25 | VSD |  |  | 2001 | 8 | VSD closure |  |  |  |  |  |  |  |  |  |  |
| 26 | VSD |  |  | 2010 | 5 | VSD closure |  |  |  |  |  |  |  |  |  |  |

*Patients receiving necessary treatment later than needed because of missing the required consultations

AS: aortic valve stenosis; ASD: atrial septum defect; AVR: aortic valve replacement; BVP: biventricular pace; CAVD: complete atrio-ventricular defect; CoA: aortic coarctation; CPB: cardio-pulmonary bypass; DCM: dilatative cardiomyopathy; DKS: Damus-Kaye-Stensel; DORV: double-outlet right ventricle; EEEA: extended end-to-end anastomosis; HLHS: hypoplastic left-heart syndrome; HRHS: hypoplastic right-heart syndrome; HTX: heart transplantation; IAVD: incomplete atrio-ventricular defect; LVAD: left ventricular assist device; mBT: modified Blalock-Taussig shunt; MS: mitral valve stenosis; MVR: mitral valve replacement; PA: pulmonary atresia; PAPVR: partial anomalous pulmonary venous return; PDA: patent ductus arteriosus; PS: pulmonary valve stenosis; PSVT: paroxysmal supraventricular tachycardy; PVR: pulmonary valve replacement; TCPC: total cavo-pulmonary connection; TGA: transposition of the great arteries; TVR: tricuspidal valve replacement; TOF: Tetralogy of Fallot; UVH: univentricular heart; VSD: ventricular septum defect
